# Oneida County

**Published:** 1897-07

**Authors:** J. M. Currie

**Affiliations:** Secretary


Secretary.

### ONEIDA COUNTY VETERINARY MEDICAL SOCIETY.

The regular quarterly meeting was held at the residence of Dr. W. G. Hollingsworth, Utica, N. Y., at 2 P.M., May 4, 1897, Dr. Hollingsworth presiding in the absence of President Dr. Morrow. There were present Drs. Hollingsworth and Skerritt, Utica; Dr. Stebbins, West Winfield; Dr. Findlay, Camden; Dr. Moore, Trenton; Dr. Markham, Port Leyden; Dr. Jones, Poland; Dr. Pratt, Herkimer; Dr. Weightman, Waterville; Drs. Huff and Currie, Rome. The minutes of the last meeting were read and approved.

Dr. E. B. Ingalls, Mohawk, and Dr. G. C. DeWitt, Ilion, were admitted to membership by a unanimous vote.

The election of officers for the ensuing year resulted as follows: President, Dr. F. Morrow, Oneida; Vice-President, Dr. Wilson Huff, Rome; Secretary and Treasurer, Dr. J. M. Currie, Rome.

During the business session articles of incorporation were signed and afterward forwarded to Albany, to be filed with the Secretary of State; a copy was also filed with the Clerk of Oneida County.

The general topic for discussion was glanders, introduced by papers by Dr. H. D. Stebbins, West Winfield, and Dr. Wilson Huff, Rome. In the animated and interesting discussion which followed (in which all members present took part), the Secretary was instructed to correspond with the State Board of Health, recommending that the mallein-test for glanders be recognized as is the tuberculin-test for tuberculosis.

The essayists were tendered a vote of thanks for their valuable contributions. There being no further business, an adjournment was in order. Next meeting to be held August 3d, in Rome, N. Y.

After closing the members present were handsomely entertained at dinner by Dr. Hollingsworth. J. M. CURRIE, V.S.,
Secretary.

